# Disclosure of mandatory and voluntary nutrition labelling information across major online food retailers in the USA

**DOI:** 10.1017/S1368980024001289

**Published:** 2024-10-17

**Authors:** Julia Reedy Sharib, Jennifer L Pomeranz, Dariush Mozaffarian, Sean B Cash

**Affiliations:** 1 Food is Medicine Institute, Friedman School of Nutrition Science and Policy, Tufts University, Boston, MA, USA; 2 School of Global Public Health, New York University, New York, NY, USA; 3 Tufts University School of Medicine, and Division of Cardiology, Tufts Medical Center, Boston, MA, USA; 4 Division of Agriculture, Food, and the Environment, Friedman School of Nutrition Science and Policy, Tufts University, Boston, MA, USA

**Keywords:** Online grocery shopping, Nutrition labelling, Food labelling regulation, Retail food environment

## Abstract

**Objective:**

Nutrition labelling is mandatory on food products in retail stores, but compliance in the rapidly expanding online setting remains unclear. We assessed mandatory and voluntary labelling information across major U.S. online retailers.

**Design:**

Between January and August 2022, we evaluated a representative basket of sixty food and beverage items across eight product categories of ten major retailers. We evaluated online presence, accessibility and legibility of four mandatory elements – Nutrition Facts, ingredients, allergen statements and percent juice for fruit drinks – and presence of seven voluntary elements – nutrient content claims, health/qualified health claims, ingredient claims, structure–function claims, additive claims, front-of-package nutrient profiling symbols and other marketing claims.

**Setting:**

Major online food retailers in the USA.

**Participants:**

N/A.

**Results:**

On average, each mandatory element was present, accessible and legible for only 35·1 % of items, varying modestly by element (from 38·3 % for ingredients lists to 31·5 % for Nutrition Facts) but widely by retailer (6·6–86·3 %). Voluntary elements were present for 45·8 % of items, ranging from 83·7 % for marketing claims to 2·0 % for structure–function claims. Findings were generally consistent across the eight product categories. Voluntary elements were more frequently present than accessible and legible mandatory elements for six of ten retailers and seven of eight product categories.

**Conclusions:**

Mandatory nutrition label elements are not commonly present, accessible and legible in online retail settings and are less consistently present than marketing elements. Coordinated industry and regulatory actions may be needed to ensure consumers can access mandatory nutrition information to make healthy and safe food choices online.

The use and reach of online grocery shopping has tremendously expanded, particularly after the onset of COVID-19. In August 2019, monthly online grocery delivery and pick-up in the United States totalled $1·2 billion. By June 2020, this number had reached $7·2 billion^(1)^. These trends are expected to continue, with anticipated annual online grocery sales of $187·7B in 2024 and a 300 % increase from 2019^(2)^. Online grocery shopping has additional implications for food and nutrition security and health equity as a result of the Supplemental Nutrition Assistance Program (a federal U.S. program that provides monthly benefits to low-income families to help them buy food) Online Purchasing Pilot, which launched in 2019 and has now been rolled out in all fifty states and DC to serve a population who, importantly, experiences disproportionately elevated rates of type 2 diabetes^(3,4)^.

The rapid adoption of online grocery shopping, however, has outpaced a similar modernisation of regulation in this space. In the physical retail setting, the U.S. FDA mandates the disclosure of specific nutrition-related information on food packages, including the Nutrition Facts label, ingredients lists, allergen statements and percent juice content for beverages purporting to contain fruit or vegetable juice^(5)^. By law, this information must appear ‘prominently and conspicuously,’ with specific requirements for what defines conspicuousness and legibility, on packaged food^(6)^. Notably, the existing regulatory language does not explicitly address the online grocery marketplace nor identify the responsible actor in that space (i.e. the manufacturer *v*. the retailer)^(7)^. In practice, the current provision of nutrition-related information in the online setting is at the discretion of the retailer who manages the marketplace.

In the absence of explicit regulation or enforcement, preliminary studies suggest important gaps in the online provision of mandated nutrition-related information. In a 2018 scan of twenty-six food items evaluated across twelve online retailers, Nutrition Facts labels and ingredient lists were present for 82 % of foods examined^(8)^. Yet, this analysis preceded the major national expansion of online food retail purchases after 2020. In a scan of fifteen foods sold at twenty-one online retailers in 2019–2020, Nutrition Facts labels were only available for 29 % of products^(9)^. A 2021 scan of ten food items across eight online retailers found that, on average, Nutrition Facts labels, ingredient lists, allergen statements and percent juice content were present, conspicuous and legible only 36·5 % of the time^(7)^. While this preliminary evidence suggests low compliance after 2019, relatively few products were evaluated, only one study was performed after the start of the COVID-19 pandemic, and all studies preceded the more recent growth and maturation of the online food retail marketplace. Thus, the extent to which major online retailers provide required nutritional information to consumers at the point of sale remains unclear.

In addition, retailers and food manufacturers have incentives to provide voluntary marketing information on products, such as health claims, nutrition content claims and ingredient claims. Such marketing claims aim to make food items more appealing to shoppers and are often displayed on food packages more prominently in comparison with mandated nutrition information. How such marketing claims are being displayed in the online grocery setting and how this compares to mandatory nutrition information are unknown.

To address these key gaps in knowledge and better understand the contemporary state of mandatory and voluntary nutrition labelling in the online setting, we investigated a comprehensive food basket of sixty common food and beverage items across eight major product categories for ten major US online retailers.

## Methods

### Selection of the food basket and online retailers

A list of sixty food and beverage items was selected with the aims of establishing a tractable and representative food basket based on the Thrifty Food Plan (a food basket developed by the USDA to estimate a cost-effective diet that meets dietary guidance, which is the basis for calculating Supplemental Nutrition Assistance Program benefit levels) and other previously evaluated food baskets identified by searching PubMed for ‘Food Basket + United States,’ ‘Thrifty Food Plan’ and ‘WIC’ (Special Supplemental Nutrition Program for Women, Infants and Children, a federal U.S. program that pays for supplemental foods for low-income pregnant, breast-feeding and postpartum women and to infants and children up to age 5). To identify a standard brand to include for each item in the basket, we used Statista.com, the Rudd Center’s Snack FACTS Report (2015) and preliminary searches to identify brands and items sold across all or most of the retailers evaluated in this study^(10)^. The resulting food basket contained items with national distribution in the USA or store-brand analogs of such items. Items were categorised across eight main product categories, based on the departments typically found in major food retailers. The full list of the sixty items and eight product categories are presented in Table 1.

**Table 1:** The standardised sixty-item food basket evaluated in this study^*^

Bakery, sweets and snacks (*n* 7)
* Bakery and sweets*
Duncan Hines Dark Chocolate Fudge Brownie Mix 18·2 oz
Hershey’s Milk Chocolate Bar, 1·55 oz
* Savory snacks*
Rold Gold Tiny Twist Pretzels, 16 oz
Triscuit Original Crackers, 8·5 oz
Pepperidge Farms Goldfish Original, 6·6 oz
* Sweet snacks*
Oreo Double Stuf Cookies, 20 oz
Quaker Chewy Chocolate Chip Granola Bars, 8 ct
Beverages (*n* 4)
* Soft drinks*
Coca-Cola Original, 12 oz cans, 24 ct
Diet Coke, 2 l bottle
* Fruit juices*
Kool-Aid Jammers Pouches, Cherry, 10 ct
Simply Orange Pulp-free 100 % Orange Juice, 52 fl oz
Dairy (*n* 8)
* Yogurt*
Chobani Greek Yogurt, Plain non-fat, 32 oz
Yoplait Original Low-fat Yogurt, Strawberry, single serve (6 oz)
* Cheese*
Kraft Singles American Cheese Slices, 16 ct
Sharp cheddar cheese, store brand, 8 oz block
* Milk*
Whole fat milk: First non-sponsored item in “Most Popular” retailer search, half gallon (64 fl oz, 1·89 l)
1 % fat milk: First non-sponsored item in “Most Popular” retailer search, gallon (128 fl oz, 3·78 l)
* Fermented milk*
Lifeway Kefir, Plain Unsweetened Low-fat Fermented Milk, 32 fl oz
* Ice cream*
Breyers Classics Natural Vanilla Ice Cream, 1·5 quart
Fats and oils (*n* 7)
Land O’Lakes Butter, salted, 1 lb (4 ct package)
Shedd’s Country Crock Original Vegetable Oil Spread, 45 oz
Hellman’s Real Mayonnaise, 30 oz Jar
Crisco Pure Vegetable Oil, 48 oz
Hidden Valley Original Ranch Dressing, 24 fl oz
Wholly Guacamole Classic Mild Guacamole, 7·5 oz
Maya Kaimal Butter Masala Simmer Sauce, 12·5 oz
Fruits and vegetables (*n* 9)
* Fruits, fresh* ^†^
Banana, 1ct
Apple, 1 ct
Strawberries, 1l b
* Fruits, frozen*
Frozen strawberries, unsweetened, 1l b
* Fruits, canned*
Store brand canned sliced peaches in juice, 15 oz
* Vegetables, fresh* ^†^
Baby carrots, store brand, 1l b
Broccoli florets, 32 oz
* Vegetables, frozen*
Store brand frozen mixed vegetables, 32 oz
* Vegetables, canned*
Store brand canned sweet whole kernel corn,15 oz
Grains (*n* 11)
* Breads*
Wonder Classic White Bread, 20 oz
Nature’s Own 100 % Whole Wheat Sliced Bread, 20 oz
Guerrero White Corn Tortillas, 30 ct (27·5 oz)
* Breakfast cereals*
General Mills Honey Nut Cheerios, 10·8 oz
Kellogg Frosted Flakes, 13·5 oz
* Ready-to-eat breakfast*
Frosted Strawberry Pop-tarts, 8 ct (13·5 oz)
* Rice*
Store Brand White Rice, 32 oz
* Pasta*
Rana Frozen Cheese Ravioli, fresh, 20 oz
Store Brand Penne Pasta, dry, 16 oz
Thai Kitchen Gluten Free Stir Fry Rice Noodles, 14 oz
* Ready-to-eat dish*
DiGiorno Rising Crust Frozen Pepperoni Pizza, 27·5 oz
Meat and eggs (*n* 9)
* Processed meat*
Oscar Mayer Deli Fresh Oven Roasted Turkey Breast, 16 oz
Hormel Black Label Original Bacon, 16 oz
Jimmy Dean Fully Cooked Original Pork Sausage Links, 12 ct (9·6 oz)
* Ready-to-eat frozen meat*
Cooked Perfect Brand Frozen Homestyle Bite Size Meatballs, 32 oz
Perdue Frozen Chicken Breast Nuggets, 20 oz
Gorton’s Frozen Crunchy Breaded Fish Fillets, 10 ct
Bibigo Frozen Korean Style Mini Wontons, Chicken & Vegetable, 24 oz
* Fresh poultry* ^†^
Fresh Chicken Bbreast, Conventional, Sstore Brand, 1 lb
* Eggs*
Eggland’s Best Large White Eggs, 1 dozen/12 ct
Plant protein (*n* 5)
Goya Dry Black Beans, 16 oz bag
Canned Black Beans, Store Brand, 15 oz can
Rosarita Traditional Refried Beans, 16 oz
Blue Diamond Whole Natural Almonds, 6 oz can
Jif Peanut Butter Creamy, Original, 40 oz Jar

* Food basket items were selected to establish a tractable and representative food basket based on the Thrifty Food Plan and other previously evaluated food baskets.

† Nutrition Facts label, ingredients list and allergen coding were omitted for fresh produce and unprocessed poultry (*n* 6 items) because these products are not mandated to display this information at the point of sale. Availability for these product/element combinations can be found in see online supplementary material, Supplemental Table S1.

Ten major online retailers, representing at least 79 % of the 2022 online grocery market in the USA^(11)^, were selected based on their involvement in the launch of the Supplemental Nutrition Assistance Program Online Purchasing Pilot, including Amazon, FreshDirect, Hy-Vee, Safeway and ShopRite (via Instacart) and/or regional or national sales prominence, including Kroger, Meijer, Publix, Stop & Shop and Walmart. Following data collection and analysis, retailers were subsequently deidentified for reporting purposes.

### Data collection

From January to August 2022, seven trained enumerators used internet browsers via MacOS and Windows to access the publicly available online sales sites for each of the ten retailers. For each retailer, the sixty items were found through searches and screenshots were taken of the full item pages, including any additional graphics or text which could only be accessed by clicking, scrolling or hovering. Dated screenshots were saved in secure, university-affiliated folders on the Box storage platform. For each search, the enumerators cleared cookies and used a browser application separate from that which was favored for personal use to prevent influence of personal browsing history on the observed web pages. If the retailer required a postal code to access item web pages, researchers entered the postal code associated with the main address of (institution redacted for review). If an exact item match (brand and package size) was not available at a given retailer, the closest analog by brand, package size or both was coded in its place and noted as a replacement.

Each item–retailer combination was coded for the presence, accessibility and legibility of four FDA-mandated elements: Nutrition Facts label, ingredients list, allergen statement and percent juice for beverages purporting to contain fruit juice^(5)^. Standards for accessibility and legibility were implemented to reflect Congressional and FDA requirements that mandatory information appear ‘prominently and conspicuously^(6)^.’ The complete coding scheme for presence, accessibility and legibility is presented in Table 2 and described further below under Analysis. If multiple versions of mandatory elements were present on the website, researchers were instructed to code for the most accessible and legible version.

**Table 2:** Coding criteria for determining the ‘availability’ of mandatory label elements

	Nutrition Facts label*	Ingredients list*	Allergens*	Percent juice‡
**Positive assessment†**				
*Presence*	Present OR abbreviated label present on packages sized 40 or fewer square inches	Present	Allergen statement present OR “no allergens” present OR common name of any major allergen present in ingredient list (e.g. “*wheat* flour”) OR ingredient list present for items containing no common allergen	Present
*Accessibility*	Fully visible on initial product page view OR visible after click on a visible item (e.g. menu or image)			
*Legibility*	Fully legible OR initially illegible but made legible with hover/zoom			
**Negative assessment**				
*Presence*	Not present OR outdated version present OR partial label/unformatted information only	Not present	Not present	Not present
*Accessibility*	Not accessible OR partially accessible OR requires scroll down/seek/click outside of initial page view			
*Legibility*	Not legible OR blurry/small (i.e. partially legible)			

* Nutrition Facts label, ingredients list and allergen coding were omitted for fresh produce and unprocessed poultry (*n* 6 items) because these products are not mandated to display this information at the point of sale. Allergen presence coding was defined in alignment with requirements set by the Food Allergen Labeling and Consumer Protection Act of 2004.

† Label elements must meet presence, accessibility and legibility criteria to be considered ‘available.’.

‡ Percent juice was assessed only for beverages that purport to contain fruit or vegetable juice (*n* 2 items).

Each item–retailer combination was also coded for the presence of seven voluntary elements regularly displayed by manufacturers, some of which must meet specific FDA requirements while others must be truthful but are less subject to regulatory oversight. These were: nutrient content claims (e.g. ‘excellent source of Ca’), health and qualified health claims (e.g. ‘Can help lower cholesterol* *Three grams of soluble fibre daily from whole grain oat foods, in a diet low in saturated fat and cholesterol, may reduce the risk of heart disease.’), structure/function claims (e.g. May support a healthy immune system as part of a balanced and healthy diet) ingredient claims (e.g. ‘contains real milk’), additive claims (e.g. ‘no artificial colors’), front of package nutrient profiling symbols (e.g. Facts Up Front) and other marketing claims (e.g. ‘Great for you!’). Definitions for all voluntary elements are provided in the supplementary materials. These elements were not evaluated for accessibility or legibility due to lack of regulatory requirements for these features when present on item packaging. The placement of voluntary elements within the main item photos *v*. webpage text was also coded to assess how retailers leverage the various spaces afforded by web pages to provide such voluntary information.

Specific coding instructions are available in this article’s supplementary materials. Coding was conducted in duplicate by two separate investigators for three retailers (Kroger, Stop & Shop and Walmart) for validation purposes, which showed high concordance (86·4 % agreement) of coding results. Any discordance was reviewed by the lead researcher by referencing screenshots of item web pages taken during the initial coding and finalised based on observations.

### Analysis

Mandatory elements were considered to be present, accessible and legible (hereafter referred to as ‘available’) if they were present on the webpage, accessible to be viewed immediately or by clicking or hovering over a feature immediately visible on the web page and legible immediately or by clicking on or hovering over an item (Table 2). Coding for Nutrition Facts labels, ingredients lists and allergens was omitted for six items (three fresh fruit items, two fresh vegetable items and one fresh unprocessed poultry item) because these products are not mandated by the FDA to display Nutrition Facts label or ingredient lists. Availability of these elements for the six products is shown in the supplementary material (see online supplementary material, Supplemental Table S1). Voluntary elements were considered present if they appeared anywhere on the webpage of each retailer–item pair, including in images or web text.

## Results

### Mandatory elements

On average across the ten retailers, the mandatory elements were available 35·1 % of the time, with generally similar average availability across the four elements: 36·5 % for ingredients lists, 35·9 % for allergens, 35·0 % for percent juice and 32·8 % for Nutrition Facts labels (Table 3). However, these findings varied substantially by retailer, with average availability of the four mandatory elements ranging from 2·4 % to 89·0 % across the individual retailers. Generally, retailers with higher compliance with any one of the elements tended to show higher compliance with the other elements as well. Across all item–retailer pairs evaluated, all mandatory label elements (two elements for fresh produce and unprocessed poultry, three for other non-juices and four for juices) were available for only 25·0 % of observations. This ranged from 0 % to 68·5 % across individual retailers.

**Table 3:** Percentage of mandatory label elements available for fifty-four food basket items across ten major online retailers in January–August 2022, by retailer and overall

	*% Available* *					
Retailer	Nutrition Facts label (*n* 54 items) †	Ingredient list (*n* 54) †	Allergens (*n* 54) †	Percent juice (*n* 2) ‡	All relevant elements§	Average availability
Retailer 1	72·2	100	98·1	0·0	**68·5**	**89·0**
Retailer 2	79·6	77·8	74·1	100	**64·8**	**77·4**
Retailer 3	70·4	66·7	55·6	100	**48·2**	**64·6**
Retailer 4	59·3	63·0	66·7	50·0	**48·2**	**62·8**
Retailer 5	18·5	29·6	29·6	50·0	**14·8**	**26·2**
Retailer 6	18·5	3·7	7·4	50·0	**0·0**	**10·4**
Retailer 7	9·3	11·1	9·3	0·0	**5·6**	**9·8**
Retailer 8	0·0	1·8	9·3	0·0	**0·0**	**3·7**
Retailer 9	0·0	7·4	5·6	0·0	**0·0**	**4·3**
Retailer 10	0·0	3·7	3·7	0·0	**0·0**	**2·4**
**Overall (across all retailers)**	**32·8**	**36·5**	**35·9**	**35·0**	**25·0**	**35·1**
se (across all retailers)	2·0	1·6	2·1	10·9	1·2	3·7

* Values represent averages by retailer; averages are calculated across all relevant items. Mandatory elements were considered available if they were present on the webpage, accessible to be viewed immediately or by clicking or hovering over a feature immediately visible on the web page and legible immediately or by clicking on or hovering over an item.

† Nutrition facts label, ingredient list and allergen coding was omitted for fresh produce and unprocessed meat (*n* 6 items) because these products are not mandated to display these label elements at point of sale.

‡ Percent juice was assessed only for beverages that purport to contain fruit or vegetable juice (*n* 2 items).

§ Percent of products for which all mandatory elements (i.e. 2, 3, or 4 elements, depending on product type) were available.

The mandatory label elements tended to have similar average availability by product category, ranging from 42·3 % for fats and oils to 29·4 % for grains (Table 4). Availability of Nutrition Facts labels was highest for fruits and vegetables (45·0 %) and lowest for meat and eggs (23·8 %). Availability of ingredient lists was highest for fats and oils (44·4 %) and lowest for bakery, sweets and snacks (30·0 %). Availability of allergens was highest for beverages (42·5 %) and lowest for bakery, sweets and snacks (28·6 %). Interestingly, Nutrition Facts labels were also available 20 % of the time for both fresh fruits and vegetables and fresh poultry (see online supplementary material, Supplemental Table S1).

**Table 4:** Percentage of mandatory label elements available for 54 food basket items across 10 major online retailers in January–August 2022, by category

	*% Available* *					
Category (number of items in each retailer basket)	Nutrition Facts label (*n* 54) †	Ingredient list (*n* 54) †	Allergens (*n* 54) †	Percent juice (*n* 2) ‡	All relevant elements §	Average availability
Fats and oils (7)	42·8	44·4	39·7	.	**33·3**	**42·3**
Fruits and vegetables (4)	45·0	44·0	40·0	.	**32·5**	**41·7**
Beverages (4)	40·0	42·5	42·5	35·0	**32·5**	**40·7**
Dairy (8)	33·8	37·5	38·8	.	**27·5**	**36·7**
Plant protein (5)	28·1	36·8	40·4	.	**19·3**	**35·1**
Bakery, sweets and snacks (7)	37·1	30·0	28·6	.	**22·9**	**31·9**
Meat and eggs (8)	23·8	37·5	33·8	.	**18·8**	**31·7**
Grains (11)	25·5	30·9	31·8	.	**21·8**	**29·4**
**Overall (across all applicable items)**	**32·8**	**36·5**	**35·9**	**35·0**	**25·0**	**35·1**
se (across all applicable items)	2·0	1·6	2·1	10·9	1·2	3·7

* Values represent averages across all retailers; averages are calculated across all relevant items. Mandatory elements were considered available if they were present on the webpage, accessible to be viewed immediately or by clicking or hovering over a feature immediately visible on the web page and legible immediately or by clicking on or hovering over an item.

† Nutrition Facts label, ingredient list and allergen coding was omitted for fresh produce and unprocessed meat (*n* 6 items) because these products are not mandated to display these label elements at point of sale.

‡ Percent juice was assessed only for beverages that purport to contain fruit or vegetable juice (*n* 2 items).

§ Percent of products for which all mandatory elements (i.e. 2, 3 or 4 elements, depending on product type) were available.

**Fig. 1:**
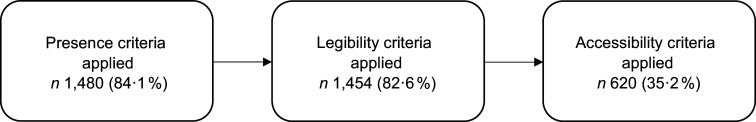
Flow chart of differences in availability in mandatory label elements across 1760 element–item–retailer combinations after stepwise application of criteria for presence, legibility and accessibility*. *Coding criteria for presence, legibility and accessibility are shown in Table 2

Across the ten retailers, mandatory elements were more frequently simply present than available (i.e. if requirements for accessibility or legibility were disregarded). On average, 84·1 % of mandatory label elements were present in some capacity on item web pages. This was similar (82·6 %) after applying legibility criteria but declined sharply to 35·2 % after applying accessibility criteria (Fig. 1). Among the four mandatory label elements, presence (regardless of accessibility or legibility) was highest for ingredient lists (98·0 %) and lowest for Nutrition Facts labels (61·9 %).

### Voluntary elements

Compared with the availability of mandatory nutrition labelling elements (35·1 %), voluntary elements were more commonly present (45·8 % of items overall) (Tables 5 and 6). One or more voluntary elements were more frequently displayed within the text of an item’s web page (88·7 % of items) than within photos of the item or its packaging (76·1 % of items) (with 71·0 % of items displaying one or more voluntary elements in both places). On average across all retailers and voluntary label elements, marketing claims were most often present (83·7 % of all items; range by retailer: 66·7–100 %), followed by nutrient content claims (49·0 %; 31·7–65·0 %) and front of package symbols (42·4 %; 28·3–56·7 %) (Table 5) – all higher than the average availability of any of the four mandatory elements. Presence of voluntary elements was lowest for health and qualified health claims (3·7 %, 0·0–8·3 %) and structure function claims (2·0 %, 0·0–3·3 %). Voluntary elements were presented most frequently by retailer 10 (50·6 %), the retailer for which mandatory information was least frequently available (4·6 %).

**Table 5:** Percentage of voluntary label elements present for sixty food basket items across ten major online retailers in January–August 2022, by retailer

	***% Present*** ^*^							
Retailer	Other/ marketing claims	Nutrient content claims	Front-of-package labels	Additive claims	Ingredient claims	Health and qualified health claims	Structure function claims	Average presence
Retailer 9	100	48·3	31·7	38·3	40·0	3·3	3·3	**50·6**
Retailer 10	93·3	51·7	51·7	26·7	36·7	3·3	1·7	**48·7**
Retailer 4	75·0	63·3	35·0	35·0	40·0	3·3	1·7	**48·2**
Retailer 8	96·7	31·7	56·7	28·3	26·7	8·3	0	**47·8**
Retailer 7	71·7	50·0	56·7	36·7	28·3	6·7	3·3	**45·7**
Retailer 1	93·3	31·7	40·0	45·0	23·3	1·7	0	**44·8**
Retailer 5	81·7	65·0	35·0	28·3	30·0	3·3	1·7	**44·6**
Retailer 6	81·7	45·0	40·0	31·7	28·3	3·3	1·7	**43·2**
Retailer 2	76·7	55·0	28·3	45·0	30·0	3·3	3·3	**42·8**
Retailer 3	66·7	48·3	46·7	33·3	38·3	0	3·3	**41·7**
**Overall (across all retailers)**	**83·7**	**49·0**	**42·2**	**34·8**	**32·2**	**3·7**	**2·0**	**45·8**
se (across all retailers)	1·5	2·0	2·0	1·9	1·9	0·8	0·6	0·7

* Values represent averages for each retailer; averages are calculated across all items. Voluntary elements were considered present if they appeared anywhere on the webpage of each retailer–item pair, including in images or web text.

**Table 6:** Percentage of voluntary label elements present for sixty food basket items across ten major online retailers in January–August 2022, by category

	*% Present* *							
Category (number of items in each retailer basket)	Other/ marketing claims	Nutrient content claims	Front-of-package labels	Additive claims	Ingredient claims	Health & qualified health claims	Structure function claims	Average presence
Dairy (8)	90·0	65·0	35·0	47·5	45·0	3·8	7·5	**53·3**
Beverages (4)	85·0	72·5	62·5	25·0	40·0	0	0	**50·2**
Grains (11)	78·2	60·9	52·7	33·6	44·6	9·1	2·7	**50·2**
Bakery, sweets and snacks (7)	90·0	25·7	67·1	45·7	45·7	1·4	0	**49·2**
Fats and oils (7)	93·7	42·9	49·2	28·6	27·0	0	1·6	**46·2**
Meat and eggs (9)	85·6	37·8	20·0	47·8	41·4	1·1	1·1	**44·0**
Plant protein (5)	80·7	59·7	38·6	15·8	5·2	10·5	1·1	**42·3**
Fruits and vegetables (9)	72·2	36·7	26·7	24·4	3·3	1·1	1·1	**32·8**
**Overall (across all items)**	**83·7**	**49·0**	**42·2**	**34·8**	**32·2**	**3·7**	**2·0**	**45·8**
se (across all retailers)	1·5	2·0	2·0	1·9	1·9	0·8	0·6	0·7

* Values represent averages across the ten major online retailers; averages are calculated across all items. Voluntary elements were considered present if they appeared anywhere on the webpage of each retailer–item pair, including in images or web text.

By product category, the average presence of voluntary elements was highest for dairy (53·5 %) and lowest for fruits and vegetables (32·8 %). The presence of voluntary elements was higher than the availability of mandatory elements for almost all categories, led by grains (absolute percentage difference of 20·8 %); bakery, sweets and snacks (17·3 %) and dairy (16·6 %) (Table 6). Only within the fruits and vegetables category were mandatory elements more frequently available (41·7 % on average) than voluntary elements (32·8 % on average), largely due to the relatively low presence of marketing claims across the products included in this category.

## Discussion

Our investigation identified that, in a scan of a standardised sixty-item basket of foods and beverages across ten online retailers, mandatory nutrition-related information was not available in most cases (35·1 %). In comparison, voluntary elements were, on average, more frequently present (45·8 %). This was particularly true for marketing claims, nutrient content claims and front of package symbols, all of which were more consistently present than any mandatory label element. Modest variation in the provision of mandatory and voluntary nutrition labelling information was present by product category, and large variation was present by retailer.

These results highlight the troubling reality that online grocery shoppers in the USA are generally not being provided access to legible, mandatory nutrition information at the point of sale. Mandatory label elements were present in some capacity for most items but were often provided in a format that was not accessible or (less frequently) not legible. Our results also confirmed that the infrastructure and technical expertise for online grocery retailers to provide this information is indeed available – all retailers in our analysis provided images and/or text descriptions in the initial page view of every item listing, both of which represent opportunities to provide mandatory nutrition information. Additionally, mandatory elements were present 85 % of the time in our analysis (regardless of accessibility or legibility), but were accessible in only 36 % of cases, meaning that the majority of these elements were only viewable by scrolling or clicking past the initial page view. In contrast, retailers often elected to display marketing graphics and text in the initial page view, rather than any of the mandatory elements. Our novel findings suggest that some retailers are actively promoting voluntary elements over mandatory nutrition information in available locations at the point of sale – a choice made possible by the current uncertain regulatory landscape around the responsible actor for online provision of nutrition information.

In addition to the common absence of accessible mandatory information, the use of voluntary nutrition claims in the food environment could perpetuate critical issues of suboptimal diet and diet-related health disparities. Suboptimal diet is a leading causes of death and disease in the USA – resulting in more than 300 000 deaths from CVD and 80 000 new cases of cancer each year^(12,13)^ – and < 7 % of all adults in the USA have optimal cardiometabolic health^(14)^. Surveys of U.S. consumers repeatedly illustrate how misleading or ambiguous language on food packaging can drive consumer confusion in the marketplace and lead to selection of less healthful food items^(15–18)^. Though many factors – including financial, cultural and taste preference – are important influences on diet, food labels also have an influence on choice, particularly in retail settings^(19)^. These relationships, when considered alongside projections for online grocery sales to continue to grow over the coming decade, make the need for clear nutrition labelling in online settings particularly poignant.

Our results support the need for modernised regulation of nutrition labelling in the online food retail space to promote standardised provision of this mandatory information to consumers. In the physical retail space, the FDA enforces regulations that require labels on packaged foods display several elements of mandatory nutrition information^(20)^. Historically, however, the retailers have not been required to carry out this mandate (although voluntary labelling regulations exist), so food manufacturers have become the party responsible for mandatory disclosures. This clarity in responsibility, however, does not carry over into the online space. Existing FDA regulations do not explicitly reference online settings where retailers, rather than manufacturers, typically manage the webpage information provided and may not embrace a role as providers of prominent and conspicuous nutrition information. The impact of this ambiguity is supported by the large variability of our results across retailers.

In 2007, the FDA issued a ‘Dear Manufacturer’ letter in which the Administration recommended the nutrition information presented online be similar to FDA’s Nutrition Facts label requirements under § 101·9^(21)^; as of 2021, this was still the FDA’s position^(22)^. FDA food labelling policy is applicable to food labelling ‘at the point of purchase^(7,20)^.’ In the case of online sales, the ‘point of purchase’ is the online webpage, suggesting the regulation applies directly to online retailers. However, recognising the need for more explicit guidance in this area, the FDA in 2023 issued a Request for Information ‘to learn more about the content, format and accuracy of food labelling information provided through online grocery shopping platforms,’ with the ultimate goal to provide further guidance on the topic^(23)^. At the time of this writing, FDA has not yet issued additional guidance or promulgated regulations on the topic.

Our study highlights the challenges consumers face in finding required nutrition information in online retail settings. At the same time, our findings also demonstrate considerable variation between retailers, suggesting such provision is feasible. Our results also point towards new opportunities to provide meaningful information online to consumers. For example, we observed that some online retailers have begun voluntarily providing nutritional information that is only recommended, but not mandatory, in physical retail establishments, such as for fresh produce^(24)^.

As the utilisation of online food shopping continues to surge, the relevance of the results and regulatory gaps we identified here may likewise grow. In addition to the relevance for the average consumer, our findings have troubling implications for individuals experiencing socioeconomic and health inequities. For example, while the expansion of the Online Purchasing Pilot to all fifty states and DC is an important step to address food access and convenience for low-income households, our results indicate that consumer protections for health information and health equity must be similarly prioritised as the retail landscape shifts.

While our findings are similar to those from studies conducted in 2018 and 2021^(7,8)^, both of which found that certain elements mandated to be on food packages by the FDA were not universally available online, our study found that this information is no more available than it was in 2021, and in fact is marginally less available (35·1 % of the time).

This study has several strengths. Our sixty-item food basket, list of retailers and label elements represent a much more comprehensive evaluation of the provision of nutrition-related information in the U.S. online food retail environment. It is also the first and only comprehensive assessment conducted in the later stages of the COVID-19 pandemic. We employed duplicate analysis in the coding of a subset of retailers and saved screenshots of food basket item web pages to enable objective validation of label element coding. We also grounded our accessibility and legibility coding criteria in current FDA regulations regarding the provision of mandatory label elements on packaged items^(6)^. Our methods for data collection and element scoring can also be used or duplicated for future studies. In sum, our findings build upon and greatly expand the observations of prior, smaller studies^(7–9)^ by diversifying the list of study retailers, food basket items and label elements. This is also the first study of its kind to evaluate and compare both mandatory and voluntary label elements for a representative food basket in the USA.

Potential limitations should be considered. Mandatory elements were primarily assessed on presence, accessibility and legibility, while voluntary elements were only assessed on presence. However, this difference in assessment was by design due to Congressional and FDA requirements that mandatory nutrition information must appear prominently and conspicuously, whereas voluntary elements have no similar requirements. The food basket and retailer selections for this study, while broad and more comprehensive compared with past studies, may not represent the findings for all food and beverage items or all online retailers. We did not evaluate small, local online retailers, which may provide greater or lesser mandatory information and voluntary elements.

In conclusion, we found that the online retail food environment is not yet providing high levels of clear and conspicuous nutrition-related information at the point of sale – information that is mandated in physical retail settings. Our findings suggest the need for coordinated industry and regulatory actions to ensure that consumers are afforded the mandatory nutritional information to make healthy and safe food choices when shopping online.

## Supporting information

Sharib et al. supplementary material Sharib et al. supplementary material

## Supplementary material

For supplementary material accompanying this paper visit https://doi.org/10.1017/S1368980024001289
