# Supplementary material for: Disclosure of mandatory and voluntary nutrition labelling information across major online food retailers in the USA
Source: Public Health Nutr. 2024 Oct 17;27(1):e203. doi: 10.1017/S1368980024001289 (PMC11604316; doi:10.1017/S1368980024001289)
Supplement: Sharib et al. supplementary material [file S1368980024001289sup001.pdf]

Supplementary Information

S1. Instructions for data collection enumerators

Data for each attribute is collected in two ways: via screenshot, and via coding sheet. Both forms of data collection must be completed for each product at each retailer. The following text details the methods for each form of data collection.

Data Collection Form 1: Product Screen Capture

The purpose of product screen capture is to record the style and content of a product's listing on a retailer's website at a given time. It is important to capture all nuances/tabs/details on these pages such that, if necessary, a team member can return to the saved screenshots and obtain the same coding results (outlined later in this document).

Product screen capture results (PDFs – see step 3) should all be saved in product-specific Box folders. If a folder with the product's name (product from food basket, *not* a product that was considered an alternative if an exact match was not available) has not yet been created, create a new folder whose name exactly matches the product description in the Food Basket document.

A master version of the template can be found here:
https://tufts.box.com/[redacted]

Steps

1) Start a new private browser session. Use a browser that you do *not* use for personal use (e.g. Firefox if you favor Chrome). Be sure that no other websites are open in the browser and clear your cookies. It is imperative that you repeat this step each day that you return to this work—do not keep the relevant browser open in the background of your other work.
2) Go to retailer website

**Note: if prompted/required to enter a zip code to access online listings, use 02155—the code for Tufts's Medford campus. There is no need to enter a zip code unless necessary.*

   a. Search for correct food/beverage – be sure to check the exact variety as well as package size. If product is found, enter "search" in the "availability" column.
   b. If the exact product is not found, do an internet search of the product + retailer name. This bypasses the restrictions based on IP address. Enter "internet" in the "availability" column.

c. If the exact product is still not found, use a different size or package option of the same brand and product. Enter “Other” in the “availability” column and record the attributes that differ from the original product in the “notes” column.

d. If a close option from the same brand is not available, enter “N/A” in the “availability” column. Repeat the process with an equivalent store brand item or the first non-sponsored item (top left) listed in the search result is to be the reference product, and enter the exact product name in the “notes” column

3) Take screen capture of entire page

a. Ensure screen zoom is at 100%

b. Take a full screenshot of the browser excluding browser items like the URL bar and bookmarks bar.

c. Click through all tabs and photos available on first page and take a full screenshot of each

i. Look for “details,” “additional information,”, “more,” “nutrition,” “ingredients,” etc.

ii. EXCLUDE taking screenshots of each square if there is a zoom in feature that only zooms to a tiny subsection

1. E.g., 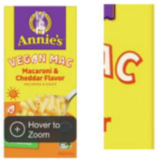

d. Scroll to next visible page and repeat

e. Repeat scroll and click through all tabs/photos

i. Exclude clicking “see more” for customer reviews or suggested products

ii. For the rest, make sure to look for “details,” “additional information,” “more,” “nutrition,” “ingredients,” etc.

f. Repeat until you reach the end of the page

g. Go to desktop and export all screenshots as PDFs

4) Compile the PDF in order of how it was screenshotted

a. Save as PDF Named store_productname_date_yourinitials

b. Upload to product specific folder within Box.

Data Collection Form 2: Nutrition Label Attributes Coding

Prior to coding, nutrition information categories were determined. Attributes for data collection are either mandatory or voluntary information for food manufacturers to provide on food labels:

Mandatory:

- Nutrition Facts Label (NFL)
- Ingredients List
- Percent Juice, when applicable
- Allergens

Voluntary:

- Health/Nutrition Claims on Package
    - Structure function claims
    - Nutrient content claims
    - Authorized and qualified health claims
    - Ingredients claims
    - Additives claims
    - Other (marketing claims)
- Health/Nutrition Claims on Website
    - Same as above
- Front-of-package labels (FOPs)

For each of the nutrition and health components, three levels for availability of website information were then created, for which each attribute will be coded for each product at each retailer:

"Presence":	Whether this feature for the specific item was available anywhere on the landing page
"Accessibility":*	Whether the information was readily available, or ease of finding the information, i.e., needing to search by scrolling or clicking
"Legibility":*	Ease of reading the information, i.e., crisp or blurry imaging or wording

** Coded for mandatory attributes only*

All attribute coding should be recorded in your copy of the coding template. A master version of the template can be found here: https://tufts.box.com/[redacted]

Code	Presence	Accessibility	Legibility
0	Not present	Not accessible	Not legible
1	Present; *for Allergens only*: Common name of major allergen(s) present on package; "No allergens" label present; allergen names absent for products without major food allergens in ingredients list. *For Nutrition Facts Label only:* Abbreviated label present on packages sized 40 or less square inches (see below)	Fully available on initial page with product	Fully legible
2	*For Nutrition Facts Label only*: Outdated or incomplete version present	Available on product page but requires click on a visible item (e.g., menu or image)	Initially illegible but made legible with hover/zoom
3		Must scroll down/seek/click; partially accessible	Blurry/small (Including partially legible)
..	NA	NA	NA
99	Error in data collection	Error in data collection	Error in data collection

Information that is not applicable to a product (e.g., "percent juice" for non-juice products, Accessibility and Legibility for voluntary attributes) is coded as ".." for non-applicable. Additionally, if any photos or other site modules suspected to provide key information about a component contained errors (e.g., broken photo link), "99" is used.

MANDATORY NUTRITION INFORMATION	CODING DETAILS
Nutrition Facts Label (NFL)	● NFP may be found in photo thumbnails in the form of a package photo or created image, in descriptions, or separate tabs/sub sections throughout the products page.<br>● Abbreviated label ("Not a significant source of ____") may be used on packages less than 40 square inches in size.<br>○ See FDA information<br>● Style of NFP should be considered. Only current style should be coded Presence=1. Outdated styles should be coded Presence=2. Side-by-side comparison of old vs. new label can be found here for reference.<br>● Partial labels or unformatted written information should be coded Presence=3. A partial label is defined as one which does not contain all information required on the new version of the NFL.<br>● If the NFL is blurry in the initial photos (coded as 1,1, 2 for Presence, Accessibility, Legibility) but a clearer label is available after scrolling or searching (coded as 1, 3, 1), final coding should be for the most legible/best version (1, 3, 1).
Ingredients	● Similar to NFP, the ingredients may be found in several areas in the form of a photo from the packaged good, or as a created image or paragraph.<br>● If the ingredients list is blurry in the initial photos (coded as 1,1, 2 for Presence, Accessibility, Legibility) but a clearer label is available after scrolling or searching (coded as 1, 3, 1), final coding should be for the most legible/best version (1, 3, 1).
Percent Juice	● This is only applicable for "juice" beverages and will often be found on the top of the NFP.<br>● For non-juice products, this category is coded as ".." for presence, accessibility, and legibility as it is not applicable.
Allergens	● Similar to health/nutrition claims, this may be listed in additional marketing photos, within copy, in or a separate section and should be coded dependent on the scenario.<br>● From FDA:<br>○ "The name of the food source of a major food allergen must appear:<br>▪ In parentheses following the name of the ingredient.<br>● Examples: "lecithin (soy)," "flour (wheat)," and "whey (milk)"<br>▪ OR immediately after or next to the list of ingredients in a "contains" statement.<br>● Example: "Contains wheat, milk, and soy."

	○ Cross contamination claims count as allergens and should be coded Presence=1.<br>▪ *Ex) "Product was produced in a facility that also processes peanuts."*<br>○ Allergen should be coded as absent if ingredient understood to include allergen is present in allergen list without presence of common allergen name<br>▪ E.g., ingredient list includes "flour," no mention of wheat; ingredient list includes "tofu," no mention of soy.

**VOLUNTARY NUTRITION INFORMATION**	**CODING DETAILS**
Health/Nutrition Claims on Packaging	• Health/nutrition claims on packaging should be determined from the provided photos/thumbnails on the website's product page only.<br>• Some packaging may be "stock photos" and may therefore not display health/nutrition claims otherwise seen in a retail setting. These cases would be coded as "0" for presence.
Health Claims on Website	• Examples include: Specific claims under sub-headers, product copy that contains claims, additional photos with claims as advertisements
Claim Types	• Structure function claims<br>• Nutrient content claims<br>○ Includes enrichment and fortification claims<br>• Authorized and qualified health claims<br>• Ingredients claims<br>○ A claim about the presence (or absence) of an ingredient, meant to convey an addition of value to the product, e.g.:<br>▪ "Made with real butter,"<br>▪ "Made with whole fruit,"<br>▪ "Contains honey"<br>▪ "100% whole wheat"<br>▪ "No high-fructose corn syrup"<br>• Additives claims<br>○ A claim about the presence or (usually) absence of an additive (artificial sweetener, flavor, color, or preservative), meant to convey an addition of value to the product, e.g.:<br>▪ "No artificial flavors"<br>▪ "No artificial sweeteners"<br>▪ "Nitrate-free"<br>• Other (marketing claims)

	○ A claim that uses other qualities of a product to encourage its purchase<br>▪ "GMO-free"<br>▪ "All-natural"<br>▪ "Gluten-free"<br>▪ "Certified vegan"<br>▪ "100% grass-fed"<br>▪ "USDA certified organic"
Front-of-package Labels (FOPs)	● FOPs may be found in image of product package or in an independent image.<br>● Unlike health claims, FOPs are icons with colors, numbers, or shapes that communicate the nutrient content of a food.<br>● The most common FOP in the U.S. food system is Facts Up Front<br>● Excludes Guiding Stars labeling scheme and other retailer-specific schemes
